# Supplementary material for: A Likelihood Approach for Real-Time Calibration of Stochastic Compartmental Epidemic Models
Source: PLoS Comput Biol. 2017 Jan 17;13(1):e1005257. doi: 10.1371/journal.pcbi.1005257 (PMC5240920; doi:10.1371/journal.pcbi.1005257)
Supplement: S1 File — (TAR.GZ) [file pcbi.1005257.s014.tar.gz › HSPH_Online-SI-Revision/EnKF07/n100-severe/n100-severe_EnKF2_table8.pdf]

1	newly 3 weeks spec	405.	295.14	{0., 1245.}	120.756	2.50808	newly 3 weeks cumul	1885.	4821.82	{532., 8011.}	174.346	0.351416	newly 5 weeks spec	212.	95.91	{0., 158.}	113.75	2.45146	newly 5 weeks cumul	2436.	5077.43	{532., 11505.}	133.635	0.305752	newly 42 weeks cumul	2940.	5239.76	{532., 12978.}	121.337	0.336114
2	newly 3 weeks spec	32.	2936.35	{11., 10430.}	9096.78	1.34302	newly 3 weeks cumul	144.	14351.5	{771., 34651.}	9870.41	1.5087	newly 5 weeks spec	7.	1221.23	{0., 3052.}	17377.3	1.76478	newly 5 weeks cumul	163.	17499.5	{975., 47751.}	10639.7	1.54533	newly 49 weeks cumul	163.	19798.5	{1184., 52550.}	12049.8	1.61486
3	newly 3 weeks spec	20.	2544.45	{22., 8045.}	12626.2	1.36647	newly 3 weeks cumul	87.	9704.93	{855., 16142.}	11055.1	1.58793	newly 5 weeks spec	9.	1361.02	{0., 4644.}	15059.6	1.6298	newly 5 weeks cumul	101.	12977.3	{860., 39026.}	12748.8	1.61228	newly 50 weeks cumul	112.	16496.8	{875., 63118.}	14629.3	1.64579
4	newly 3 weeks spec	173.	2813.21	{131., 13080.}	1542.97	0.932568	newly 3 weeks cumul	880.	10314.	{2452., 38779.}	1072.82	0.774729	newly 5 weeks spec	105.	2034.98	{21., 8298.}	1874.04	0.991228	newly 5 weeks cumul	1112.	14782.1	{2590., 57986.}	1231.19	0.789422	newly 48 weeks cumul	1341.	22938.2	{2601., 98533.}	1613.11	0.82315
5	newly 3 weeks spec	780.	1564.29	{0., 2888.}	211.804	2.17743	newly 3 weeks cumul	2856.	19747.3	{5657., 61511.}	591.431	0.618064	newly 5 weeks spec	402.	185.64	{0., 654.}	91.5821	2.10297	newly 5 weeks cumul	3744.	20406.8	{5664., 61872.}	445.054	0.517433	newly 42 weeks cumul	4829.	20613.2	{5664., 61881.}	326.864	0.413224
6	newly 3 weeks spec	252.	2792.04	{0., 9035.}	1074.07	1.67191	newly 3 weeks cumul	1205.	15036.	{3372., 19092.}	1150.12	0.853979	newly 5 weeks spec	97.	1966.28	{0., 6214.}	1992.85	1.77711	newly 5 weeks cumul	1441.	19352.8	{4800., 43435.}	1245.16	0.896833	newly 48 weeks cumul	1575.	26476.8	{4955., 90011.}	1582.98	0.999972
7	newly 3 weeks spec	63.	1330.63	{246., 1201.}	2019.95	0.980993	newly 3 weeks cumul	435.	5885.15	{1304., 5200.}	1259.16	0.837969	newly 5 weeks spec	20.	578.72	{59., 589.}	2804.4	1.13246	newly 5 weeks cumul	497.	7341.15	{1562., 6643.}	1383.71	0.871564	newly 49 weeks cumul	509.	8569.9	{1722., 7863.}	1590.35	0.931491
8	newly 3 weeks spec	292.	484.98	{17., 865.}	152.808	0.672275	newly 3 weeks cumul	1253.	4419.78	{596., 11588.}	269.655	0.432876	newly 5 weeks spec	140.	237.64	{0., 519.}	181.471	1.5872	newly 5 weeks cumul	1606.	4985.28	{596., 11693.}	233.153	0.425608	newly 45 weeks cumul	1911.	5737.39	{596., 11801.}	227.965	0.454561
9	newly 3 weeks spec	0.1	2576.04	{122., 5096.}	2.57594×10^{6}	3.97403	newly 3 weeks cumul	4.	13736.9	{871., 26993.}	343323.	3.21415	newly 5 weeks spec	0.1	1145.24	{17., 1948.}	1.14514×10^{6}	3.3747	newly 5 weeks cumul	4.	16570.4	{1008., 32035.}	414161.	3.28655	newly 50 weeks cumul	4.	19821.9	{1036., 36216.}	495449.	3.3515
10	newly 3 weeks spec	115.	810.04	{0., 2004.}	661.357	1.09277	newly 3 weeks cumul	507.	10416.5	{2715., 11952.}	1961.69	1.09317	newly 5 weeks spec	26.	329.35	{0., 594.}	1244.58	1.31292	newly 5 weeks cumul	597.	11226.7	{3331., 16509.}	1786.08	1.0536	newly 43 weeks cumul	631.	13258.5	{3391., 34188.}	2002.57	1.06912
11	newly 3 weeks spec	53.	1316.02	{247., 1128.}	2383.06	1.01422	newly 3 weeks cumul	267.	4791.38	{1121., 4516.}	1694.52	0.99926	newly 5 weeks spec	7.	733.64	{57., 682.}	10380.9	1.51247	newly 5 weeks cumul	288.	6530.78	{1370., 5923.}	2167.63	1.06535	newly 49 weeks cumul	300.	8392.06	{1557., 7788.}	2697.35	1.13724
12	newly 3 weeks spec	467.	507.24	{0., 1349.}	142.176	2.49263	newly 3 weeks cumul	2017.	14314.2	{6888., 14001.}	614.323	0.698035	newly 5 weeks spec	256.	88.6	{0., 179.}	97.3594	2.38681	newly 5 weeks cumul	2654.	14606.4	{7082., 14464.}	455.294	0.595307	newly 42 weeks cumul	3292.	14682.1	{7085., 14555.}	351.14	0.509929
13	newly 3 weeks spec	476.	199.18	{0., 274.}	119.517	3.05297	newly 3 weeks cumul	2172.	6250.2	{2620., 6289.}	192.645	0.276274	newly 5 weeks spec	224.	21.38	{0., 17.}	97.8571	2.89339	newly 5 weeks cumul	2758.	6328.31	{2620., 6465.}	137.515	0.196605	newly 42 weeks cumul	3330.	6360.14	{2620., 6495.}	111.626	0.182044
14	newly 3 weeks spec	744.	422.26	{0., 989.}	111.777	2.64035	newly 3 weeks cumul	3264.	8488.48	{3216., 8237.}	169.432	0.236254	newly 5 weeks spec	407.	67.48	{0., 140.}	94.0688	2.6516	newly 5 weeks cumul	4260.	8717.2	{3239., 8408.}	127.735	0.207124	newly 42 weeks cumul	5829.	8778.69	{3239., 8417.}	105.463	0.262928
15	newly 3 weeks spec	170.	1207.42	{11., 2364.}	655.8	0.70687	newly 3 weeks cumul	891.	14146.5	{1633., 48750.}	1490.41	0.852303	newly 5 weeks spec	60.	196.45	{0., 249.}	332.75	1.20431	newly 5 weeks cumul	1068.	14764.5	{1789., 49186.}	1284.99	0.801882	newly 44 weeks cumul	1164.	15018.2	{2422., 49189.}	1192.61	0.781129
16	newly 3 weeks spec	648.	808.48	{25., 1229.}	110.966	0.613286	newly 3 weeks cumul	2744.	6535.7	{426., 7500.}	174.028	0.409534	newly 5 weeks spec	394.	335.74	{0., 395.}	113.751	1.07694	newly 5 weeks cumul	3600.	7359.48	{584., 7862.}	145.407	0.376726	newly 43 weeks cumul	4481.	10230.4	{909., 32708.}	172.951	0.372843
17	newly 3 weeks spec	33.	1793.48	{197., 3990.}	5337.15	1.38949	newly 3 weeks cumul	309.	7577.43	{1107., 14026.}	2357.68	1.11093	newly 5 weeks spec	37.	948.69	{74., 1680.}	2469.86	1.03165	newly 5 weeks cumul	396.	9818.68	{1252., 20365.}	2384.9	1.11427	newly 49 weeks cumul	405.	12958.3	{1342., 33928.}	3104.61	1.21294
18	newly 3 weeks spec	122.	4567.97	{1278., 13874.}	3644.24	1.3501	newly 3 weeks cumul	687.	19099.5	{5984., 56457.}	2680.13	1.23771	newly 5 weeks spec	37.	2618.36	{482., 8013.}	6976.65	1.52714	newly 5 weeks cumul	830.	25165.3	{7430., 79429.}	2931.96	1.28035	newly 47 weeks cumul	928.	33044.7	{8768., 103160.}	3460.85	1.34528
19	newly 3 weeks spec	77.	2203.85	{533., 7789.}	2764.64	1.18759	newly 3 weeks cumul	381.	6767.89	{2004., 20319.}	1679.56	1.07017	newly 5 weeks spec	32.	1987.65	{252., 8384.}	6112.66	1.36271	newly 5 weeks cumul	445.	10873.2	{2923., 36965.}	2346.28	1.15455	newly 47 weeks cumul	467.	22530.4	{4196., 86481.}	4726.79	1.33007
20	newly 3 weeks spec	99.	1143.86	{27., 2714.}	1103.39	0.682332	newly 3 weeks cumul	531.	7620.58	{2577., 13915.}	1335.14	0.937766	newly 5 weeks spec	19.	420.88	{0., 1091.}	2213.47	1.34991	newly 5 weeks cumul	601.	8693.52	{2582., 21428.}	1346.51	0.920261	newly 45 weeks cumul	622.	11114.8	{2583., 38671.}	1686.94	0.948232
21	newly 3 weeks spec	24.	1351.37	{228., 933.}	5532.04	1.33743	newly 3 weeks cumul	229.	4634.04	{1369., 4187.}	1926.02	1.0715	newly 5 weeks spec	14.	863.61	{62., 562.}	6071.36	1.18577	newly 5 weeks cumul	254.	6606.27	{1655., 5125.}	2502.82	1.11819	newly 49 weeks cumul	256.	9038.94	{1809., 7234.}	3432.37	1.19023
22	newly 3 weeks spec	57.	2498.4	{632., 8402.}	4283.16	1.40026	newly 3 weeks cumul	329.	16280.4	{4194., 67139.}	4848.46	1.48731	newly 5 weeks spec	27.	402.51	{79., 789.}	1397.96	1.10155	newly 5 weeks cumul	392.	17634.2	{4496., 71836.}	4398.52	1.46173	newly 47 weeks cumul	426.	18117.9	{5216., 72152.}	4153.03	1.45092
23	newly 3 weeks spec	0.1	1053.36	{62., 855.}	1.05326×10^{6}	3.52682	newly 3 weeks cumul	12.	6431.73	{228., 4088.}	53497.8	2.29631	newly 5 weeks spec	0.1	434.15	{17., 301.}	434050.	2.92423	newly 5 weeks cumul	12.	7521.88	{322., 4784.}	62582.3	2.36179	newly 51 weeks cumul	12.	8520.52	{532., 5607.}	70904.3	2.41641
24	newly 3 weeks spec	10.	3574.29	{344., 19691.}	35642.9	2.08707	newly 3 weeks cumul	103.	17163.4	{3219., 78057.}	16563.4	1.92769	newly 5 weeks spec	0.1	633.55	{18., 2479.}	633450.	3.38462	newly 5 weeks cumul	103.	19300.5	{3851., 88157.}	18638.3	1.98939	newly 52 weeks cumul	103.	20124.5	{4255., 89353.}	19438.4	2.03328
25	newly 3 weeks spec	0.1	3060.78	{40., 12555.}	3.06068×10^{6}	3.59085	newly 3 weeks cumul	33.	11005.8	{1605., 45407.}	33250.8	2.0829	newly 5 weeks spec	0.1	946.22	{1., 2740.}	946134.	2.667	newly 5 weeks cumul	33.	13856.1	{1611., 75234.}	41888.2	2.13789	newly 52 weeks cumul	33.	15327.7	{1646., 82069.}	46347.5	2.17056
26	newly 3 weeks spec	105.	3515.6	{266., 11459.}	3249.9	1.15606	newly 3 weeks cumul	559.	19685.7	{4016., 64761.}	3422.39	1.35361	newly 5 weeks spec	34.	1174.05	{7., 2054.}	3382.97	1.14543	newly 5 weeks cumul	657.	22878.6	{4467., 77762.}	3383.24	1.33466	newly 46 weeks cumul	704.	24670.7	{4641., 85180.}	3405.37	1.32874
27	newly 3 weeks spec	67.	2536.56	{241., 6074.}	3685.91	1.28106	newly 3 weeks cumul	373.	8816.94	{771., 13450.}	2263.79	1.1338	newly 5 weeks spec	19.	1879.13	{229., 8549.}	9790.16	1.59726	newly 5 weeks cumul	421.	12867.9	{1246., 29654.}	2956.51	1.22199	newly 47 weeks cumul	444.	21564.7	{4030., 89067.}	4756.91	1.37299
28	newly 3 weeks spec	219.	3715.24	{295., 10523.}	1602.62	0.879336	newly 3 weeks cumul	956.	21164.2	{1845., 64632.}	2117.27	0.976198	newly 5 weeks spec	119.	1182.23	{69., 3801.}	912.798	0.739808	newly 5 weeks cumul	1227.	24438.7	{2117., 70149.}	1895.49	0.942636	newly 46 weeks cumul	1619.	26136.4	{2289., 71599.}	1518.94	0.871546
29	newly 3 weeks spec	122.	4039.45	{569., 9149.}	3211.02	1.3374	newly 3 weeks cumul	698.	13161.4	{1791., 21715.}	1785.59	1.13408	newly 5 weeks spec	37.	3023.17	{382., 10173.}	8070.73	1.66811	newly 5 weeks cumul	790.	19738.1	{2765., 42453.}	2398.49	1.24005	newly 47 weeks cumul	845.	31957.2	{6112., 93347.}	3681.92	1.40228
30	newly 3 weeks spec	189.	749.	{0., 1791.}	357.81	1.19851	newly 3 weeks cumul	1013.	5282.17	{1547., 5817.}	425.421	0.569698	newly 5 weeks spec	86.	424.82	{0., 1832.}	496.907	1.31515	newly 5 weeks cumul	1247.	6243.64	{1909., 9581.}	404.674	0.537876	newly 43 weeks cumul	1455.	11184.8	{2225., 52020.}	672.686	0.574322
31	newly 3 weeks spec	1.	3675.29	{167., 23514.}	367429.	2.905	newly 3 weeks cumul	19.	12488.6	{2522., 60828.}	65629.5	2.49772	newly 5 weeks spec	0.1	890.03	{9., 4900.}	889930.	3.17982	newly 5 weeks cumul	19.	15344.1	{2863., 78990.}	80658.2	2.56032	newly 52 weeks cumul	19.	16354.	{2999., 82217.}	85973.4	2.59864
32	newly 3 weeks spec	28.	2287.77	{195., 10903.}	8078.11	1.4254	newly 3 weeks cumul	154.	12974.7	{3577., 26817.}	8325.1	1.6183	newly 5 weeks spec	6.	994.06	{4., 2036.}	16483.	1.49763	newly 5 weeks cumul	176.	15471.2	{3902., 54719.}	8690.44	1.61547	newly 50 weeks cumul	190.	17188.4	{3988., 84099.}	8946.52	1.61077
33	newly 3 weeks spec	32.	1865.64	{235., 3927.}	5730.13	1.45069	newly 3 weeks cumul	204.	14482.4	{3208., 24300.}	6999.22	1.57082	newly 5 weeks spec	18.	378.31	{28., 508.}	2005.72	1.01793	newly 5 weeks cumul	235.	15699.	{4418., 24745.}	6580.44	1.54927	newly 50 weeks cumul	268.	16030.1	{5292., 24782.}	5881.37	1.50646
34	newly 3 weeks spec	218.	536.04	{0., 1099.}	231.211	1.11746	newly 3 weeks cumul	1079.	11510.6	{5559., 11740.}	970.131	0.884323	newly 5 weeks spec	118.	58.76	{0., 130.}	71.6102	1.49736	newly 5 weeks cumul	1346.	11741.5	{5777., 12297.}	775.797	0.801865	newly 42 weeks cumul	1517.	11774.9	{5781., 12361.}	679.726	0.753564
35	newly 3 weeks spec	0.1	2878.34	{89., 10291.}	2.87824×10^{6}	3.95033	newly 3 weeks cumul	24.	15308.9	{3926., 55126.}	63687.1	2.51422	newly 5 weeks spec	0.1	899.92	{2., 2572.}	899832.	3.21001	newly 5 weeks cumul	24.	17818.6	{3980., 63631.}	74144.	2.57467	newly 51 weeks cumul	24.	19001.9	{3980., 63651.}	79074.4	2.60608
36	newly 3 weeks spec	53.	859.25	{241., 1113.}	1522.7	0.911419	newly 3 weeks cumul	336.	3409.45	{915., 4759.}	916.176	0.804347	newly 5 weeks spec	17.	445.93	{64., 503.}	2525.	1.02728	newly 5 weeks cumul	377.	4492.49	{1337., 5960.}	1092.82	0.845946	newly 49 weeks cumul	391.	5550.97	{2153., 7621.}	1320.71	0.906928
37	newly 3 weeks spec	3.	1592.29	{221., 1340.}	52976.3	2.39897	newly 3 weeks cumul	35.	9975.23	{3740., 7998.}	28400.7	2.20773	newly 5 weeks spec	0.1	483.67	{14., 612.}	483570.	3.19834	newly 5 weeks cumul	35.	11292.3	{4052., 8325.}	32163.8	2.26146	newly 51 weeks cumul	35.	12140.8	{4154., 8633.}	34588.1	2.29018
38	newly 3 weeks spec	40.	1228.64	{227., 2245.}	2975.05	1.18352	newly 3 weeks cumul	256.	6191.58	{1354., 9591.}	2322.07	1.09396	newly 5 weeks spec	12.	511.91	{73., 1112.}	4169.75	1.29913	newly 5 weeks cumul	282.	7492.39	{1592., 12168.}	2560.37	1.13134	newly 49 weeks cumul	298.	8522.14	{1683., 14818.}	2763.28	1.15805
39	newly 3 weeks spec	150.	3341.49	{380., 8673.}	2130.1	1.05912	newly 3 weeks cumul	734.	18980.7	{2653., 61955.}	2487.72	1.11387	newly 5 weeks spec	40.	1117.28	{97., 2698.}	2694.55	1.12968	newly 5 weeks cumul	880.	22006.7	{2935., 67482.}	2402.68	1.10905	newly 46 weeks cumul	975.	23796.3	{3027., 69039.}	2342.65	1.10809
40	newly 3 weeks spec	92.	1670.57	{114., 2407.}	1724.21	1.07188	newly 3 weeks cumul	452.	7374.55	{440., 10522.}	1541.9	1.03684	newly 5 weeks spec	39.	830.48	{81., 1350.}	2036.62	1.13539	newly 5 weeks cumul	559.	9374.83	{602., 13166.}	1586.3	1.04984	newly 49 weeks cumul	609.	11728.5	{1147., 15068.}	1833.83	1.11257
41	newly 3 weeks spec	14.	1121.71	{325., 1484.}	7913.5	1.62581	newly 3 weeks cumul	127.	7358.16	{3098., 7653.}	5693.83	1.55577	newly 5 weeks spec	4.	369.51	{40., 828.}	9146.25	1.57055	newly 5 weeks cumul	135.	8332.2	{3305., 8469.}	6072.	1.58413	newly 51 weeks cumul	136.	9310.38	{3353., 11217.}	6745.87	1.61558
42	newly 3 weeks spec	202.	2123.87	{2., 3483.}	1039.79	1.07441	newly 3 weeks cumul	987.	16437.1	{2109., 68810.}	1570.78	0.764497	newly 5 weeks spec	95.	476.26	{0., 203.}	520.926	1.6706	newly 5 weeks cumul	1233.	17897.1	{2422., 76044.}	1357.98	0.699222	newly 44 weeks cumul	1344.	18377.9	{2545., 76045.}	1274.27	0.673368
43	newly 3 weeks spec	252.	1809.89	{0., 9008.}	676.845	1.36308	newly 3 weeks cumul	1153.	11904.6	{3198., 30614.}	937.002	0.817819	newly 5 weeks spec	99.	890.24	{0., 5604.}	874.646	1.30642	newly 5 weeks cumul	1387.	14057.3	{3979., 43511.}	916.568	0.777342	newly 43 weeks cumul	1536.	16221.8	{3979., 58873.}	956.487	0.760123
44	newly 3 weeks spec	39.	1473.37	{108., 2136.}	3681.41	1.29402	newly 3 weeks cumul	267.	10624.9	{1291., 10395.}	3882.97	1.3149	newly 5 weeks spec	9.	532.59	{4., 1149.}	5833.	1.39993	newly 5 weeks cumul	301.	11996.8	{1669., 11472.}	3889.14	1.3329	newly 50 weeks cumul	304.	13178.2	{2136., 14129.}	4238.36	1.38074
45	newly 3 weeks spec	130.	4154.62	{0., 9799.}	3145.35	1.73025	newly 3 weeks cumul	816.	17707.7	{3524., 22048.}	2070.06	1.06882	newly 5 weeks spec	64.	2930.52	{0., 11825.}	4528.94	1.82944	newly 5 weeks cumul	987.	24116.5	{5966., 46679.}	2343.41	1.1353	newly 48 weeks cumul	1048.	35449.3	{7246., 91235.}	3282.56	1.2809
46	newly 3 weeks spec	72.	1707.86	{609., 2642.}	2273.17	1.22534	newly 3 weeks cumul	478.	7119.01	{2469., 10730.}	1390.86	1.04597	newly 5 weeks spec	30.	948.26	{290., 1833.}	3061.67	1.29594	newly 5 weeks cumul	550.	9354.16	{3321., 13350.}	1602.22	1.09763	newly 47 weeks cumul	566.	12345.2	{4273., 21715.}	2082.45	1.19451
47	newly 3 weeks spec	463.	1037.18	{22., 2822.}	240.505	0.813671	newly 3 weeks cumul	1902.	8665.5	{2319., 37338.}	358.984	0.40821	newly 5 weeks spec	209.	238.56	{0., 264.}	165.656	1.85524	newly 5 weeks cumul	2424.	9400.54	{2336., 40248.}	292.467	0.327053	newly 43 weeks cumul	2941.	9664.09	{2336., 40359.}	241.542	0.288913
48	newly 3 weeks spec	166.	2287.03	{0., 4934.}	1333.73	1.62925	newly 3 weeks cumul	968.	13826.1	{2822., 19205.}	1330.6	0.907553	newly 5 weeks spec	51.	1732.86	{0., 7926.}	3353.76	1.71669	newly 5 weeks cumul	1120.	17546.1	{3689., 38971.}	1466.62	0.947303	newly 48 weeks cumul	1215.	25898.5	{4650., 86269.}	2031.56	1.05817
49	newly 3 weeks spec	3.	3903.9	{235., 19770.}	130030.	2.62325	newly 3 weeks cumul	31.	19379.4	{3699., 78264.}	62414.1	2.50302	newly 5 weeks spec	0.1	801.29	{10., 2560.}	801190.	3.32886	newly 5 weeks cumul	32.	21877.7	{4648., 88409.}	68267.7	2.55377	newly 52 weeks cumul	32.	23702.5	{5456., 89736.}	73970.4	2.60655
50	newly 3 weeks spec	18.	1383.67	{317., 1260.}	7587.06	1.56985	newly 3 weeks cumul	46.	9486.69	{3168., 8769.}	20523.2	2.04399	newly 5 weeks spec	1.	368.65	{22., 568.}	36767.	2.15726	newly 5 weeks cumul	52.	10533.4	{3493., 9383.}	20156.6	2.0431	newly 51 weeks cumul	52.	11528.6	{3550., 13310.}	22070.3	2.07943
